# Case Report: A Case Report on an 18-Year-Old Female with Cerebral Vasculitis in Systemic Lupus Erythematosus (SLE).

**DOI:** 10.12688/f1000research.152704.1

**Published:** 2024-06-11

**Authors:** Sakshi Dudhe, Gaurav V Mishra, Pratap Singh Parihar, Devyansh Nimodia, Dhananjay Shinde, Anjali Kumari

**Affiliations:** 1 Department of Radio-Diagnosis,, Datta Meghe Institute of Higher Education and Research, Wardha, Maharashtra, 442001, India

**Keywords:** treatment challenges, multiorgan failure, immunological dysfunction, neurological complications, systemic lupus erythematosus (SLE), cerebral vasculitis

## Abstract

Cerebral vasculitis is a rare but severe complication of Systemic Lupus Erythematosus (SLE), presenting significant challenges in management due to its potential for devastating neurological consequences and poor prognosis. We present a case of an 18-year-old female with known SLE who presented with seizures, declining cognitive function, and unresponsiveness. Neurological examination, laboratory investigations, and radiological imaging supported the diagnosis of cerebral vasculitis secondary to SLE. Despite aggressive immunosuppressive therapy, the patient’s neurological status continued to deteriorate, leading to respiratory failure and multiorgan dysfunction. Ultimately, the patient succumbed to multiorgan failure attributed to severe CNS vasculitis and its complications. This case underscores the importance of early recognition and aggressive management of cerebral vasculitis in SLE while highlighting the need for further research into more effective therapeutic strategies to improve patient outcomes.

## Introduction

Systemic Lupus Erythematosus (SLE) is a chronic autoimmune disease characterized by immune dysregulation, leading to inflammation and tissue damage in multiple organs and systems. Though mostly SLE primarily affects women of childbearing age, its clinical presentation can vary widely, ranging from mild symptoms to life-threatening complications. ^1^ Neurological manifestations occur in approximately 50% of SLE patients during the course of their illness, with varying degrees of severity and clinical presentations. ^2^ Cerebral vasculitis, although rare, is one of the most severe neurological complications of SLE, leading to significant morbidity and mortality. ^3^ SLE impacts numerous organ systems, such as the muscles and peripheral and central neurological systems. While involvement of the central nervous system (CNS) is widespread in SLE patients, resulting in a variety of neuropsychiatric symptoms, cerebral vasculitis is uncommon; postmortem investigations have shown that its frequency is less vasculitis in SLE is characterized by inflammation of the blood vessels supplying the brain, resulting in impaired blood flow, ischemia, and tissue damage. This condition can manifest clinically as seizures, focal neurological deficits, cognitive impairment, and altered consciousness. ^4^ Diagnosing cerebral vasculitis in SLE often requires clinical assessment, laboratory investigations, and neuroimaging studies. Laboratory findings may include positive antinuclear antibodies (ANA), elevated inflammatory markers, and evidence of multiorgan involvement. ^5^ Neuroimaging studies such as magnetic resonance imaging (MRI) are crucial in detecting cerebral vasculitis-related abnormalities, including white matter lesions, infarcts, and haemorrhages. ^6^ Treatment of cerebral vasculitis in SLE typically involves aggressive immunosuppressive therapy aimed at suppressing the underlying autoimmune process and reducing inflammation. Corticosteroids, cyclophosphamide, and other immunomodulatory agents are commonly used to achieve disease control and prevent further neurological deterioration. ^7^ Despite advances in the management of SLE and its complications, cerebral vasculitis remains a challenging condition associated with high rates of morbidity and mortality. Early recognition, prompt intervention, and close monitoring are essential to optimize outcomes in affected patients. ^8^ In this context, we present a case report of an 18-year-old female with SLE who developed cerebral vasculitis, highlighting the clinical features, diagnostic challenges, and management strategies associated with this rare but severe complication of the disease.

## Case presentation

An 18-year-old female patient, previously diagnosed with Systemic Lupus Erythematosus (SLE) one year back, presented to the emergency department with episodes of seizures characterized by clenching of the jaw and abnormal movements of the upper and lower limbs, accompanied by severe headache, declining cognitive function, and decreased responsiveness for 6 hours. For one year she was a known SLE case and in between this time period, she had symptoms like painful joints, fatigue, malar rashes, photosensitivity, oral ulcers and hair loss. There was no notable family history of autoimmune diseases or neurological disorders.

During the general physical examination in Emergency Department, her blood pressure was measured at 140/80 mm Hg, with a pulse rate of 84 bpm and oxygen saturation of 98%. The neurological assessment revealed generalized tonic-clonic seizures, limited spontaneous movements, and non-reactive pupils. Deep tendon reflexes were found to be sluggish to absent. Systemic examination of Cardiovascular system, Respiratory system and Gastrointestinal system yielded normal findings. An erythematous malar rash, sparing the nasolabial folds, consistent with SLE, was observed.

### Diagnostic assessment and treatment

The patient underwent comprehensive laboratory and radiological investigations, revealing significant findings, including positive Anti Smith antibody (Anti Sm) which is the most specific antibody in SLE and Antinuclear antibody (ANA) which is most sensitive antibody in SLE, with low complement levels (C3, 51 mg/dL; C4, 6 mg/dL). She was under suspicion of presenting with iron deficiency anemia (Hb 4.6 gm%), leading to the prescription of iron protein succinylate oral solution for a duration of 4.5 weeks; however, there was no improvement was noted. A blood smear analysis revealed an elevated quantity of spherocytes, suggesting the presence of an autoimmune hemolytic mechanism that necessitated the administration of corticosteroid therapy.

Leukocytosis and thrombocytopenia were noted, likely caused by ongoing infection or immunosuppressive therapy drugs. Elevated serum creatinine levels (4.1 mg/dL), which was indicative of poor kidney function was also a finding. Arterial blood gas analysis indicated metabolic acidosis (serum bicarbonate 10 mEq/L, pH 7), blood lactate levels were 4.5 mmol/requiring bicarbonate correction. Cerebrospinal fluid (CSF) analysis demonstrated elevated protein levels and pleocytosis, indicative of CNS inflammation.

**Figure 1.**
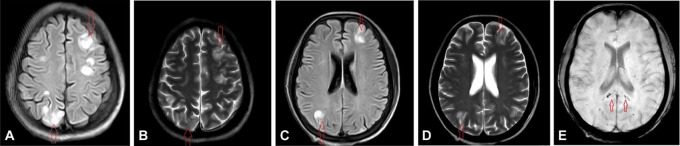
A) MRI Brain-FLAIR sequences showing hyperintensity in the left frontal and right parietal region, B) MRI Brain-T2 hyperintensity in left frontal and right parietal region, C) MRI Brain-FLAIR sequence showing hyperintensity in the left frontal and right parietal region at the level of lateral ventricles, D) MRI Brain-T2 hyperintensity in left frontal and right parietal region, E) MRI Brain-SWI sequence showing petechial hemorrhages in splenium of corpus callosum.

Radiological examinations comprised abdominal and pelvic ultrasonography and MRI Brain imaging. Ultrasonography revealed increased ecotexture of kidneys with loss of cortico-medullary junction, suggestive of chronic kidney disease. MRI Brain, conducted on a 3 Tesla MRI machine, revealed T2WI/FLAIR hyperintensities in the cerebral cortex and subcortical white matter in the left frontal, bilateral parietal, and high parietal regions. Additionally, multiple diffusely scattered petechial hemorrhages were noted in the bilateral cerebral lobes, predominantly involving the splenium of the corpus callosum and bilateral internal capsule as shown in Figure 1.

The patient received treatment including antiepileptics (in. Levipil 1 gm IV BD) for seizure control, corticosteroids (in. Methylprednisolone 1 gm IV OD) for inflammation control, and cyclophosphamide (in. Cyclophosphamide) according to the Eurolupus protocol to address the ongoing autoimmune process. Given persistently falling blood pressure, intravenous Norad was administered. Despite aggressive medical intervention for SLE, the patient’s neurological status continued to deteriorate. She developed respiratory failure with pulmonary edema, necessitating ventilation support. Despite maximal supportive care, she remained comatose and unresponsive, ultimately succumbing to multiorgan failure secondary to severe CNS vasculitis and its complications, including seizures, with SLE considered as the attributing cause of death.

## Discussion

Cerebral vasculitis is a rare but severe complication of Systemic Lupus Erythematosus (SLE), characterized by inflammation of the brain’s blood vessels. It presents a significant challenge in management due to its potential for devastating neurological consequences and poor prognosis. ^9^ In this case, the patient presented with a myriad of neurological symptoms, including seizures, declining cognitive function, and unresponsiveness, indicative of CNS involvement in SLE. The presence of a malar rash, positive Antismith antibody (Anti Sm), Antinuclear antibody (ANA), and other laboratory findings consistent with SLE further supported the diagnosis. Additionally, radiological imaging revealed findings of cerebral vasculitis, including T2WI/FLAIR hyperintensities and petechial hemorrhages in the brain parenchyma. ^10^ However, secondary infections needs to be ruled out using contrast enhanced MRI. In addition to conventional MRI, vessel wall MRI (VW-MRI) may be helpful in differentiating between vascular narrowing caused by intracranial atherosclerotic disease (ICAD) plaque, reversible cerebral vasoconstriction syndrome, dissection, and moyamoya disease, as well as vasculitis, which is characterized by contrast enhancement of the affected arterial wall. Treatment strategies for cerebral vasculitis in SLE typically involve aggressive immunosuppressive therapy to suppress the underlying autoimmune process and reduce inflammation. In this case, the patient received a combination of corticosteroids and cyclophosphamide, to control disease activity and prevent further neurological deterioration. ^11^ However, despite these interventions, the patient’s neurological status continued to deteriorate, highlighting the challenges in managing this complication of SLE. The development of subsequent respiratory failure and multiorgan dysfunction further compounded the complexity of the case with classic SLE findings. Despite maximal supportive care of this known SLE patient, including ventilation support and hemodynamic management, the patient succumbed to multiorgan failure, CNS vasculitis and its complications. ^12^

## Conclusions

In conclusion, the presented case underscores the formidable challenge of cerebral vasculitis in the context of Systemic Lupus Erythematosus (SLE). Despite aggressive medical intervention, including immunosuppressive therapy and supportive care, the patient’s neurological status continued to deteriorate, ultimately leading to multiorgan failure and a fatal outcome. This case highlights the limitations of current treatment approaches and the urgent need for further research into more effective therapeutic strategies for managing cerebral vasculitis in SLE. Additionally, it emphasizes the importance of early recognition and prompt initiation of treatment to improve outcomes in such cases. Ultimately, the complexity and severity of cerebral vasculitis in SLE underscore the necessity for a multidisciplinary approach involving rheumatologists, neurologists, and critical care specialists to optimize patient care and mitigate the devastating consequences of this condition.

## Disclosure

Human subjects: Consent was obtained or waived by all participants in this study.

## Consent to publish

Written informed consent for publication of their clinical details and images was obtained from the patient, who volunteered to participate in this study and gave permission for this study. She was explained the possible risks and benefits of this study and provided adequate information concerning the study in her language.

## Data Availability

No data are associated with this article.
