# Supplementary material for: Case Report: A Case Report on an 18-Year-Old Female with Cerebral Vasculitis in Systemic Lupus Erythematosus (SLE)
Source: F1000Res. 2024 Jun 11;13:614. [Version 1] doi: 10.12688/f1000research.152704.1 (PMC11372350; doi:10.12688/f1000research.152704.1)
Supplement: Peer review history

# Reviewer report: Reviewer response for version 1

**DOI:** 10.5256/f1000research.167497.r313640

**Published:** 2024-09-03

**Authors:** Ke Xu

**Affiliations:** 1 Third Hospital of Shanxi Medical University, Taiyuan, China

This article is scientifically and clinically sound. Cerebrovascular inflammation is a severe neurological complication of systemic lupus erythematosus (SLE), making this patient particularly significant for clinicians, especially those specializing in rheumatology and immunology. The case involves a young female with SLE presenting with acute onset symptoms, requiring aggressive treatment that eventually led to neurological deterioration. A comprehensive physical examination of the patient remains necessary, emphasizing the importance of clinician vigilance. Furthermore, considering the patient's one-year history of SLE, it is crucial to explore her medication regimen during this period and evaluate its efficacy. Additionally, details regarding the duration and implementation of this treatment plan are still required. A full supplementary explanation is recommended. In addition, the references cited are old, and new advances in diagnosis and treatment remain to be fully elucidated.

Are enough details provided of any physical examination and diagnostic tests, treatment given and outcomes?

Partly

Is the case presented with sufficient detail to be useful for other practitioners?

Yes

Is sufficient discussion included of the importance of the findings and their relevance to future understanding of disease processes, diagnosis or treatment?

Yes

Is the background of the case’s history and progression described in sufficient detail?

Yes

Reviewer Expertise:

Clinical Rheumatology Immunology

I confirm that I have read this submission and believe that I have an appropriate level of expertise to confirm that it is of an acceptable scientific standard.

## References

[1] : Central Nervous System Systemic Lupus Erythematosus: Pathophysiologic, Clinical, and Imaging Features. *Radiographics* .2022;42(1) : 10.1148/rg.210045 212-232 10.1148/rg.210045 34990324

# Reviewer report: Reviewer response for version 1

**DOI:** 10.5256/f1000research.167497.r298294

**Published:** 2024-08-28

**Authors:** Angel Justiz-Vaillant

**Affiliations:** 1 University of the West Indies, St. Augustine, Trinidad and Tobago

The article is scientifically sound and very interesting. It will be served to other practitioners in the field of rheumatology and/or immunology to manage a case like this one. The workout of SLE was done correctly and clinically there is not doubt that we are in front of an interesting case of lupus. I invite the readers to access it by themselves.

Are enough details provided of any physical examination and diagnostic tests, treatment given and outcomes?

Yes

Is the case presented with sufficient detail to be useful for other practitioners?

Yes

Is sufficient discussion included of the importance of the findings and their relevance to future understanding of disease processes, diagnosis or treatment?

Yes

Is the background of the case’s history and progression described in sufficient detail?

Yes

Reviewer Expertise:

Clinical Immunology

I confirm that I have read this submission and believe that I have an appropriate level of expertise to confirm that it is of an acceptable scientific standard.

## References

[1] : Neuropsychiatric Systemic Lupus Erythematosus: Molecules Involved in Its Imunopathogenesis, Clinical Features, and Treatment. *Molecules* .2024;29(4) : 10.3390/molecules29040747 10.3390/molecules29040747 38398500 PMC10892692

# Response

**Published:** 2024-08-28

**Authors:** Sakshi Dudhe

**Affiliations:** Radiodiagnosis, Not Applicable, Sawangi,Wardha, Maharashtra, India

Thank you!
